# GH/IGF-1 Signaling and Current Knowledge of Epigenetics; a Review and Considerations on Possible Therapeutic Options

**DOI:** 10.3390/ijms18101624

**Published:** 2017-10-05

**Authors:** Francisco Álvarez-Nava, Roberto Lanes

**Affiliations:** 1 Biological Sciences School, Faculty of Biological Sciences, Central University of Ecuador, Quito 999165, Ecuador; 2 Pediatric Endocrine Unit, Hospital de Clínicas Caracas, Caracas 999188, Venezuela; lanesroberto@gmail.com

**Keywords:** Growth Hormone, Insulin-like Growth Factor I, epigenetics, gene regulation, intrauterine growth retardation, epigenetic drugs, metabolism, postnatal growth

## Abstract

Epigenetic mechanisms play an important role in the regulation of the Growth Hormone- Insulin-like Growth Factor 1 (GH-IGF1) axis and in processes for controlling long bone growth, and carbohydrate and lipid metabolism. Improvement of methodologies that allow for the assessment of epigenetic regulation have contributed enormously to the understanding of GH action, but many questions still remain to be clarified. The reversible nature of epigenetic factors and, particularly, their role as mediators between the genome and the environment, make them viable therapeutic target candidates. Rather than reviewing the molecular and epigenetic pathways regulated by GH action, in this review we have focused on the use of epigenetic modulators as potential drugs to improve the GH response. We first discuss recent progress in the understanding of intracellular molecular mechanisms controlling GH and IGF-I action. We then emphasize current advances in genetic and epigenetic mechanisms that control gene expression, and which support a key role for epigenetic regulation in the cascade of intracellular events that trigger GH action when coupled to its receptor. Thirdly, we focus on fetal programming and epigenetic regulation at the *IGF1* locus. We then discuss epigenetic alterations in intrauterine growth retardation, and the possibility for a potential epigenetic pharmaceutical approach in short stature associated with this fetal condition. Lastly, we review an example of epigenetic therapeutics in the context of growth-related epigenetic deregulation disorders. The advance of our understanding of epigenetic changes and the impact they are having on new forms of therapy creates exciting prospects for the future.

## 1. Introduction

Epigenetic marks are inherited from one cell to another by meiosis or mitosis, but are also quasi-stable, and, consequently, reversible. Understanding these epigenetic modifications will allow us to know what is normal and what is abnormal. However, the reversible nature of epigenetic factors opens up the possibility of modifying them and restituting the normal phenotype. Recent research has focused on the epigenetic modifications of the Growth Hormone- Insulin-like Growth Factor I (GH-IGF-I) axis, as epigenetic mechanisms are involved in the regulation of the GH-IGF-I axis and in processes controlling long bone growth, carbohydrate and lipid metabolism, liver metabolic function, and energy balance. Improvement of methodologies that assess epigenetic regulation has contributed enormously to the understanding of GH action, but many questions still need to be clarified. The quasi-stable, heritable and reversible nature of epigenetic factors and, particularly, their role as mediators between the genome and the environment make them stimulating therapeutic target candidates.

In this review, we emphasize advances in the knowledge and comprehension of the role of epigenetic regulation in gene expression of the GH-IGF-I axis brought about by recent research, and how this relates to our current understanding of the mechanisms by which growth-related disorders arise. We also discuss the substantial evidence for an altered GH-IGF-I axis in intrauterine growth retardation (IUGR), which has been associated with short stature and metabolic disorders. It is likely that the development of pathological epigenetic modifications of the GH-IGF-I axis is involved in the evolution of these conditions. By modifying the epigenetic marks responsible for an altered fetal environment, it may be possible for us to also potentially diminish the risk for the appearance of such conditions. This epigenetic approach allows for the integration of an ever-growing repertoire of epigenetic signatures with alternative forms of therapy.

## 2. Intracellular Signals Regulating Growth Hormone Actions

Growth Hormone (GH) is an anterior pituitary-derived 191 amino acid-long single chain hormone, which is important in the promotion of long bone growth, the regulation of carbohydrate and lipid metabolism, liver metabolic function and energy balance. It is synthesized, stored and secreted by the somatotroph cells within the anterior pituitary gland. Its secretion is dependent on a complex network of intracellular and extracellular signals that regulate hormone synthesis and release. At the cellular level, GH acts to regulate cell growth, differentiation, apoptosis, and reorganization of the cytoskeleton. The effects of this protein hormone can be mediated in an indirect manner through Insulin-like Growth Factor 1 (IGF-I), a 70 amino acid-long circulating peptide, which is produced in the liver and other tissues in response to GH (IGF-I-dependent GH effects). These indirect effects include regulation of growth, glucose uptake and protein metabolism. Growth hormone action can also involve more direct transcriptional responses in target tissues (IGF-I independent GH effects) such as stimulating insulin secretion, lipolysis, and gluconeogenesis [1].

The more than 400 actions of GH are mediated by an array of signals triggered by its activated cell surface receptor, known as Growth Hormone Receptor (GHR). This is a 620 amino acid-long single transmembrane protein in target tissues. The binding of GH-GHR results in activation of several intracellular signaling pathways and expression of a diverse set of genes, which enables GH to exert its pleiotropic effect. These events are predominantly mediated by the Janus-Family Tyrosine Kinase-2 (JAK2). Activation of JAK2 leads to mitogenic proliferation, recruiting and/or phosphorylation of a variety of intracellular signaling molecules, including Signal Transducers and Activators of Transcription (STAT)-1, -3 and -5), Mitogen-Activated Protein Kinases (MAPKs), Insulin Receptor Substrate-1 (IRS1), Phosphatidylinositol- 3-Phosphate-Kinase (PI3K), Diacylglycerol (DAG), Protein Kinase-C (PKC), intracellular calcium (Ca^++^), and induction of *IGF-1* and other growth hormone-dependent genes.

The GH molecule binds to one subunit of GHR, after which the GH-GHR complex contacts a second subunit GHR to form a 1:2 trimolecular stoichiometric complex (GH-(GHR)_2_), leading to receptor activation [2]. It is known that the GH binding sites on the extracellular domains of the two subunits are placed asymmetrically. However, a new model for GHR activation based on a relative rotation of cytoplasmic subunits within a constitutive homodimer has been described [3] (see below). This is based on the finding that the two subunits of the GHR are constitutively dimerized in an inactive (i.e., unbound) state [4]. In addition, the two monomers of GHR are joined by their transmembrane domains through leucine zipper interactions, with steric hindrance from extracellular domains preventing interactions between identical receptor partners [5].

The GHR is a type 1 glycoprotein and a member of the superfamily of transmembrane proteins that includes the prolactin receptor and a number of cytokine receptors. Like other cytokine receptor superfamily members, the GHR has an extracellular domain (ECD) of 246 amino acids, a single transmembrane domain (TCD), and an intracellular domain (ICD). The C-terminal region of the GHR is required for tyrosine phosphorylation of the receptor, and for a hormonal effect on gene transcription, whereas only 46 membrane proximal amino acids of the cytoplasmic domain are necessary for the activation of JAK2 and the transduction of the GH proliferative signal. The interaction of GH with GHR is mediated by two asymmetric binding sites on GH. Site 1 has a somewhat higher affinity than Site 2 to GH, and Site 1 mediates the first binding step. The GHR is likely dimerized even in the absence of the ligand, as is observed for other Class I cytokine receptors. The GH-GHR complex has three binding interfaces: GH binding Site 1 with an extracellular Domain 1, GH binding Site 2 with an extracellular Domain 2, and the so called “dimerization interface” between the second subdomains of extracellular Domain 1 and the extracellular Domain 2. This last interaction is stronger than the GH-extracellular Domain 2. GH binding Site 1 and the formation of the dimerization interface are not functionally coupled. GH binding might result in a clockwise rotation of the receptor JAK2 binding region (via the juxtamembrane transmembrane domain) torsion that brings the JAK2 molecules into proximity for kinase transphosphorylation. This model suggests that both ECD and ICD juxtamembrane domains of the GHR adopt a rigid conformation to allow the transmission of the torque force resulting from the asymmetrical binding of GH. However, the mechanism by which GH binding converts the inactive predimerized GHR to its active signaling conformation is uncertain.

GH-induced homodimerization of the GHR appears to be a prerequisite for biological activity of the hormone as receptor dimerization precedes signal transduction. The dimerized GHR then relays its signal by recruiting cytoplasmic tyrosine kinases, which phosphorylate tyrosine residues in the ICD of GHR and induce downstream signaling events. There is evidence that a specific cytoplasmic domain of GHR mediates JAK2 activation, metabolic actions of GH, STAT activation and calcium influx. Some authors also suggest that receptor predimerization is mediated by the TCD and that GH binding initiates signaling by triggering changes in the orientation of the two GHRs within the dimer [6].

### 2.1. GH Signal Transduction Pathway

After the binding of GH to its receptor, JAK2 is activated and becomes bound to a conserved proline-rich sequence of the cytoplasmic domain of GHR close to the cell membrane. Thus, hormone-dependent dimerization brings these two kinases into proximity [7,8,9]. Upon recruitment, the activated JAK2 molecule causes phosphorylation of critical tyrosines within the kinase-activation loop on the intracellular portion of the GHR, a sort of transphosphorylation, hence inducing JAK2 activation. These phosphorylated tyrosines on the receptor provide docking sites for recruiting critical intermediary STAT proteins.

Although seven mammalian STATs have been described, STAT5b appears to be most critically involved in mediating the growth-promoting actions of the GHR [3]. However, most transcripts that are increased with GH signaling are not dependent upon STAT5b, since only 20% of GH-regulated transcripts declined in abundance in the setting of a dominant-negative STAT5b [10]. As a member of the STAT family of proteins, which possess a SRC Homology 2 (SH2) domain and a conserved tyrosine residue near the C-terminus, STAT5b requires tyrosine phosphorylation on ligand-activated GHR for its activation as JAK2 phosphorylation of selected receptor cytoplasmic domain tyrosine residues sets up binding sites for the SH2 domain. Thus, STAT5b is recruited to the GHR-JAK2 complex, and also becomes tyrosine phosphorylated [11,12]. This aligns bound JAK2 and Src kinases, and their activation by transphosphorylation results in a cascade of signaling events. Further phosphorylation of STAT proteins at serine residues is followed by their dimerization and dissociation from the GHR, and then translocation to the nucleus. Thus, STAT proteins serve as direct signal transducers to the nucleus that can activate gene transcription by binding to defined DNA response elements adjacent to target genes [13,14,15]. The binding of STAT dimers to interferon γ-activated sites (GAS) on target genes results in the induction of transcription of these genes.

Ultimately, the growth-promoting effects of GH are determined by hormone-induced modifications in IGF1 gene expression since IGF1 is the major, but not the only, mediator of GH action on somatic growth [16]. Also, transcription of several other genes such as *c-fos* and *c-jun* [5,17,18], and the *Serine protease inhibitor*, *Spi 2.1* [19], are acutely activated by GH.

### 2.2. Intracellular Signals Regulating Growth Hormone Actions

The *IGF1* gene is composed of six exons and five introns that span a genomic range of greater than 80 kb, generating over 100 distinct IGF1 mRNAs [20]. To achieve this, several tandem- promoters regulate the gene expression of *IGF1* through a unique leader exon, but involve several transcription start sites with multiple ATG codons at the open reading frame. Thus, Exon 1 or Exon 2 can be spliced to Exon 3. Consequently, Exon 1-derived (Promoter 1 (P1)) transcripts are expressed in multiple tissues and use different transcription initiation sites [20,21]. Instead, Promoter 2 (P2) is smaller and simpler than P1 and Exon 2-derived (P2) transcripts are mainly but not exclusively, expressed in hepatic cells [21]. Furthermore, hepatic IGF1 mRNA also involves the inclusion or exclusion of Exon 5, leading to changes in the translational reading frame of the amino-acid coding region of Exon 6 [22,23]. Thus, the IGF1A transcript lacks Exon 5, while IGF1B transcript contains Exon 5. Moreover, multiple polyadenylation sites in the 3′untranslated region (UTR) of the *IGF1* gene transcribe different high-molecular-weight mRNAs (from 0.8 to 7.5 kb) [20,21]. The activated GHR is able to immediately stimulate both *IGF1* promoters, and triggers the transcription of all classes of IGF1 mRNAs. In spite of so many transcripts only one of two IGF-I protein precursors (either IGF1A or IGF1B) and one single 70-amino acid mature IGF-I peptide is encoded. Several hypotheses have been put forward to explain these findings [20]. First, it has been proposed that each class of IGF1 mRNA has characteristic features, such as distinct half-lives [24]. Another hypothesis is that a range of mRNA turnover rates guarantees a sustained IGF1 production after gene activation is induced possibly through the presence of a group of IGF1 RNAs that are insensitive to different microRNAs or other inhibitory molecules [25,26].

Recent studies have established that GH controls the production of IGF-I by powerfully activating the gene transcription of *IGF1* through STAT5b in response to diverse physiological stimuli mediated by GH [20,27]. Canonical sequences of STAT5 binding sites in both promoters of the *IGF1* have not been identified [19]. Instead, GH-induced binding of STAT5b has been identified within each proximal promoter region of the *IGF1* locus [19]. This finding is similar to those found in other genes activated by GH such as *CISH*, *SOCS2*, *IGFALS* and *SPI2.1*. In addition, studies using the chromatin immunoprecipitation (ChIP) methodology have not detected Stat5b binding sites within either promoter [27,28,29,30]. In contrast, this technique has demonstrated multiple Stat5b binding domains dispersed through the *IGF1* locus. Thus, some of the STAT5b binding domains within the *IGF1* locus may be transcriptional enhancers for the *IGF1* gene or may act as decoys that bind STAT5b and sequester it from activating sites. Similarly, paired binding sequences have been found in the proximal regions of the promoters of the other Stat5b-regulated genes, including *CISH*, *SOCS2*, *IGFALS* and *SPI2.1* [19]. The existence of other transcriptional response elements in the *IGF1* gene is likely, since studies with ChIP only focus on conserved and paired sequences. Therefore, GH-activated *IGF1* gene transcription may be stimulated by binding of STAT5b in chromatin to multiple dispersed conserved transcriptional response elements. The affinity and transcriptional power of these elements have been demonstrated to vary significantly between different DNA response elements and there is no close correlation between affinity and transcriptional potency [19]. Consequently, the binding of multiple dispersed enhancers of STAT5b may exert combinatorial control over the activity of the *IGF1* gene.

It is probable that GH mediated STAT5b binding elements at the *IGF1* locus provoke mono-methylation of lysine 4 on histone H3 (H3K4), and by binding of p300, Med I, and RNA polymerase II as a similar mechanism of STAT1 by interferon γ has been described [31,32,33]. However, it is also possible that some GH-induced STAT5b binding chromosomal elements are not truly transcriptional enhancers of the IGF1 locus, but rather may act in a transient manner as decoys that bind STAT5b and sequester it from activating sites [20]. Three mechanisms of action may be hypothetically appealed to, to regulate *IGF1* gene expression via STAT5b. First, STAT5b can directly activate the gene transcription on *IGF1* by interacting with dispersed transcriptional DNA response elements in the IGF1 locus, which act as transcriptional enhancers [27]. Secondly, the binding of STAT5b to some transcriptional DNA response elements in the *IGF1* locus may facilitate a physical association between two distal genomic regions in chromatin to active *IGF1* gene transcription [34]. Lastly, STAT5b may indirectly regulate *IGF1* gene transcription by a reduction in the action and amount of transcription repressors [19,35,36]. For example, in the absence of GH activated signaling, Bcl6, a transcriptional repressor known to have DNA recognition sequences resembling that of Stat5b, is bound to the Stat5b element in *Socs2*, *Cish* and *Spi2.1* promoters [19]. This binding is reverted when GH treatment is used, leading to a rapid replacement of BCL6 with STAT5b at these sites [19]. Thus, *Bcl6* gene transcription is inhibited by GH via STAT5b.

### 2.3. Epigenetic Regulation of the GH-IGF-I Axis

Epigenetics is the study of quasi-stable, reversible and heritable modifications of chromatin or DNA structure (and their consequent phenotypic expression) involved in the regulation of gene transcription that are not attributable to change in the DNA sequence. Thus, epigenetic effects, i.e., phenotypic expression, can be stable enough to be inherited from one cell to its progeny, somatic, or germ-line cells, and can also be changed (reversible). However, they differ from genetic traits in that they are not encoded by the DNA sequence. The genomic configuration (chromatin structure) and locations of these modifications may alter cell- and tissue-specific patterns of gene expression (epigenetic regulation) and be mediated by the interaction of the genome with a variety of environmental factors. Therefore, gene expression is regulated by reversible modifications of the eukaryote genetic material to generate changes in the patterns of gene transcription in response to these environmental factors. Consequently, an epigenetic pathway implicates the response and processing of external signals, resulting in adjustments that change and maintain specific patterns of gene expression. Because epigenetic changes are quasi-stable, potentially reversible, and influenced by environmental factors, the knowledge of molecular mechanisms of epigenetics opens the possibility for the development of drugs for the treatment of conditions linked to the dysfunction of an epigenetic pathway.

Most evidence suggests that epigenetic modifications are carried out by four mechanisms: (1) DNA methylation of CpG sites; (2) post-translational covalent modifications of histones (histones code) and the use of variant histone proteins; (3) remodeling of nucleosomes and/or reorganization of chromatin on a larger scale; and (4) regulation of gene expression by small, noncoding RNA molecules. These modifications are reversible, changing the chromatin configuration (open or closed), activating or silencing genes, and do not occur in isolation, i.e., the overall effect is obtained by the combination, type, site and extent of modifications. Thus, the chromatin, in which the transcriptionally active genes reside, is in a lax (relaxed) state to allow access for RNA polymerase II and associated factors. The open configuration is obtained by the combination of epigenetic modifications such as unmethylated (hypomethylated) CpG islands [37,38], lysine acetylation at histone 4 (H4Kac) and methylation of lysine 4 residues at histone 3 (H3K4) [39]. On the other hand, the low histone acetylation, (hyper)methylation of CpG sites, and trimethylation (me3) of H3K9 and H3K27 (H3K9me3 and H3K27me3, respectively) are associated to closed silenced chromatin, which prevents access of the transcriptional machinery (RNA polymerase and associated factors) [40,41,42,43,44].

It is conceivable that a suitable fetal environment for postnatal growth could be achieved by "priming" and maintaining open chromatin around the *IGF1* locus through GH and other pituitary factors, so that in postnatal life the GH-IGF-I axis expression gene is facilitated.

#### 2.3.1. Fetal Programming and Epigenetic Regulation at the *IGF1* Locus

Serum IGF1 levels largely depend on hepatic IGF-I production and exhibit a developmental epigenetic regulation that is modulated by GH through multiple GH response elements, (GHREs) [20,27,30,45,46,47] which interact with multiple tandem binding sites of varying transcriptional potency in the *IGF1* locus. Conservation nucleotides and amino acids of IGF-I in human and rat species, as well as a comparable expression of multiple mRNA variants [46], have been noted for both species. For a marked increase of IGF1 mRNA levels to occur, it is necessary that the gene transcription machinery has access to the hepatic *IGF1* Promoter 1 region. Consequently, chromatin remodeling at this locus is essential. Following the second week of life in the rat, a significant rise in hepatic IGF-1 mRNAs occurs, concurrent with a progressive increase in accessible chromatin at Intron 2 Growth Hormone Response Element (IN2GHRE) locus, demonstrated by vulnerability to DNase I digestion [47]. IN2GHRE is a potent enhancer of *Igf1* gene transcription and is in turn upregulated by STAT5b, another GHRE [20,27,30,46]. Thus, upon GH activation, STAT5b mediates GH induction of hepatic IGF-I almost exclusively [48,49]. At the eighth week of life, IN2GHRE demonstrates complete vulnerability to DNase I digestion. In addition, GH administration to adult hypophysectomized rats increases H3K4 trimethylation (H3K4 me3) around IN2GHRE without affecting either promoter [45]. H3K4me3 [50,51] and H3K36me3 [52] are epigenetic marks associated with open, accessible and active chromatin, i.e., actively transcribed gene regions. This early developmental epigenetic maturation pattern may be essential for the maintenance of an optimal GH-IGF1 axis during infancy, childhood, adolescence and adult life and can be altered by Intrauterine Growth Retardation (IUGR).

Certain early post-natal events may influence *IGF1* gene expression through epigenetic modifications. It is conceivable that a favorable fetal environment for optimal expression of the GH-STAT5b-IGF-I pathway could involve not only the activation of the GH-GHR complex, but also of other pituitary trophic hormones during the early stages of postnatal development. This assumption could be viable, given that the chromatin around the inactive promoters of *IGF1* gene is open [45]. Absence of epigenetic modifications that keep the chromatin open around *IGF1* promoters in the fetal period may explain the postnatal deterioration of the GH-IGF-I axis seen in IUGR.

Intrauterine growth retardation is defined as a fetal growth rate of less than 10 percent of predicted fetal weight for gestational age, which may result in significant fetal and neonatal morbi-mortality. Any maternal, fetal, or placental alteration may potentially affect biological activity in the fetus and can lead to growth failure. There is evidence for an association between IUGR and short stature in adulthood [53]. Data have also shown that IUGR may be associated with an adult life increased prevalence of metabolic syndrome, which is related to obesity, hypertension, Type 2 diabetes mellitus (T2DM), and cardiovascular disease [54]. Alterations in the GH-IGF-I axis seem to be a common pathophysiological feature shared by all these associations.

Intrauterine growth retardation affects systemic GH-IGF1 homeostasis in both humans and rats by disrupting the epigenetic regulation of hepatic *IGF1* gene transcription and normal developmental increases in hepatic IGF1 mRNAs. IUGR is associated with decreases in circulating IGF1 levels, hepatic *IGF1* mRNA variants, and H3K36me3 at the hepatic *IGF1* locus [55]. In addition, IUGR interferes with the normal developmental accessible chromatin at the hepatic *Igf1* IN2GHRE site as it alters the epigenetic pattern of H3K4me3 and H3K36me3 and DNA CpG methylation around this locus [45]. Thus, IUGR changes the developmental pattern of histone modifications of the GHREs, and affects the epigenetic profile of hepatic *Igf1* along its entire length. Many of these epigenetic changes persist postnatally from the perinatal period into adult life associated with decreased hepatic Igf1 mRNA and serum protein levels [55]. Furthermore, while the hepatic *IGF1* histone code does not vary between genders under normal conditions, the postnatal effect of IUGR on histone modifications spanning the hepatic *Igf1* locus are different between males and females [55]. This stable and gender-specific epigenetic pattern offers insight into possible mechanisms through which IUGR may predispose the appearance of adult life disorders, which are prevalent in males. Thus, IUGR might induce changes in epigenetic developmental programming that lead to late-life pathological conditions, given that they disrupt normal developmental epigenetic marks, and consequently, the expression of hundreds of GHRE-containing genes with diverse functions.

These findings in IUGR rat models, which contain an evolutionarily similar *IGF* locus gene constitution to humans, can be only extrapolated in a speculative manner to human pathophysiology. However, it could assist in deciphering the epigenetic regulation of human *IGF1* gene expression, which could be useful in the diagnosis of growth alterations in Small for Gestational Age (SGA) subjects, and for the management and rational use of rhGH therapy. For example, rhGH therapy at supraphysiological doses is known to improve the final height of SGA subjects. Knowledge of the regulatory epigenetic pattern of the human *IGF1* locus would help identify an accessible chromatin around the *IGF1* locus. This may lead to the use of a lower dose of rhGH in SGA subjects with fewer adverse side effects, opening the possibility of a more personalized medicine.

Several therapeutic targets could be considered in growth promoting therapy. An adjuvant therapy would promote histone code elements that maintain open chromatin around the *IGF1* gene and/or reverse DNA methylation, H3-K9 methylation and the low histone acetylation, which are pathologically established modifications by an altered fetal environment. This potential therapy would likely be supplemental to rhGH. It could also have additional effects on growth promotion such as those seen following GH therapy in IUGR rats where it causes a dramatic and rapid increase in the levels of core histone acetylation with a 20-fold rise being detected at two *Igf1* promoters within 60 min of systemic hormone administration [19]. Therefore, adjuvant therapy to rhGH in SGA patients that stimulates growth via promotion of open chromatin induced by code histone or reversal of closed chromatin via DNA methylation or low acetylation and methylation of H3-K9 would be supplementary, would not substitute for rhGH and would maximize its effectiveness, reduce the dose of rhGH needed, increasing its tolerance, and decreasing its toxicity and side effects of rhGH.

#### 2.3.2. Epigenetic Alterations in Intrauterine Growth Retardation Open the Possibility for a New Pharmaceutical Approach in Short Statured Small for Gestational Age Subjects

Epigenetic drugs are a relatively new class of pharmacological agents acting on chromatin enzymatic and non-purely enzymatic complexes [42], Epigenetic therapy has been comprehensively studied in cancer, and several epigenetic drugs are being used therapeutically in the control of malignant diseases. However, this therapy is currently reaching far beyond the initial field of oncology, and has recently emerged as an alternative approach in the development of therapeutic strategies for nonmalignant diseases.

Several drugs have been described to target epigenetic marks, and effectively reverse DNA methylation and histone modifications. Most of these pharmacological agents directly target the epigenetic signatures through inhibition of DNA Methyl Transferase (DNMT) and Histone Deacetylase (HDAC) enzymes. DNMT inhibitors rapidly reactivate the transcription of genes that have undergone silencing via promoter DNA methylation, particularly if this silenced gene expression has arisen as a consequence of a pathological condition. 5-azacytidine (5-Aza) and its deoxy-analog (5-Aza-deoxy-cytidine) were the first DNA methylation inhibitors approved by the US Food and Drug Administration (FDA). These nucleotide analogs are incorporated into replicating DNA in place of cytidine (cytosine) nucleotides, leading to the formation of demethylated DNA [56,57]. Another cytidine analog, zebularine, has been demonstrated to cause demethylation and reactivation of a silenced and hypermethylated gene [58]. Also, DNMT inhibitors have now effectively been combined with HDAC inhibitor agents, another class of drugs that target epigenetic marks [59]. HDAC inhibitors interfere with the catalytic domain, and thereby block substrate recognition and induce gene expression. The FDA has so far only approved one HDAC inhibitor, Varinostat.

The epigenetic marks that distinguish hepatic or chondrocyte *IGF1* loci in IUGR from their normal counterparts are also reversible. Studies of epigenetic regulation of the GH-IGF1 axis in rats with IUGR suggest that large numbers of CpG in GHRE loci are differentially methylated, so that the normal histone signature pattern is altered [45,55]. Therefore, epigenetic pharmacological agents might have the potential of reversing the aberrant gene expression modifications observed in IUGR and consequently re-establish normal molecular pathways. Thus, administration of drugs that reverse epigenetic alterations might lead to global re-expression of previously silenced genes. However, some genes that are re-expressed following epigenetic drugs may not be methylated before intervention, and others that may be expressed before drug treatment might show reduced expression [60,61]. These facts reflect the reversal of epigenetic silencing of an upstream regulator that could either stimulate or repress its downstream targets. In addition, epigenetic drugs may affect various other classes of substrates, including molecules in signaling pathways and cellular architecture [42]. Consequently, several aspects need to be considered regarding genome-wide acting epigenetic enzyme inhibitors. Moreover, further studies to elucidate the biochemical mechanisms responsible for promoting and maintaining a GH-mediated open chromatin around and within the *IGF1* locus, are necessary. For example, it is known that histone acetyl-transferases and the transcriptional co-activator p300 play a role in post-translational histone modifications. However, it is probable that other unidentified histone acetyl-transferases are responsible for gene expression activation of *IGF1*, since p300 is less abundant at Promoter 2 than at Promoter 1 of the *IGF1* gene [19].

Combining DNMT and HDAC inhibitors has been suggested as an approach for epigenetic therapy, as a synergistic effect of this combination has been described in the hypermethylated gene locus [62]. A strategy in growth promoting therapy could theoretically be a combination of a DNMT inhibitor and rhGH, with or without a HDAC inhibitor. This synergistic effect of epigenetic drugs could theoretically increase the sensitivity of rhGH, leading to a decrease in the dose used and consequently, fewer adverse collateral effects. Epigenetic modulator drugs may also have the therapeutic potential for reducing the risk of insulin resistance that has been described in many individuals receiving rhGH. However, the wide spectrum of epigenetic modifications that regulate gene transcription in the GH-IGH1 axis, have only recently begun to be unraveled. In spite of the growing understanding of the role of epigenetic dysregulation as cause-and/or-effect for the metabolic syndrome of IUGR, various aspects are still not well comprehended, and require additional research. Thus, further study of specific epigenetic drugs acting on the GH-IGF-I axis is required. It will be imperative to distinguish the specific molecular epigenetic regulatory pathways and the epigenetic mechanisms that govern the GH-IGF1 axis and to identify other biomarkers and candidate genes. In addition to targeting DNA methylation and the histone code, or treatment with DNMT and HDAC inhibitors, approaches targeting miRNA expression will need to be explored in the context of IUGR.

Prader-Willi Syndrome (PWS) is an example of epigenetic deregulation of a GH-related phenotype due to a deficiency of paternally expressed genes. Normally, the differential methylation of CpG islands [63] along with acetylation of histone H3 lysine 4 (H3K4) and the methylation of histone H3 lysine 9 (H3K9) [64,65] on maternal PWS-associated genes is consistent with the activation of paternal genes of the 15q11–q13 chromosomal region, a phenomenon known as genomic imprinting (parental monoallelic expression). Gene expression at this imprinted chromosomal region is controlled by a regulatory element known as a PWS imprinting centre (PWS-IC). A cell-based high-content screen identified small molecules (UNC0638 and UNC0642) previously characterized to be selective inhibitors of Euchromatic Histone Lysine N-Methyltransferase 2 (EHMT2; also known as G9a) [66]. Both UNC0638 and UNC0642 derepressed (activated) a maternal PWS associated gene known as Small Nuclear Ribonucleoprotein N Polypeptide (SNRPN), which is a PWS-IC-controlled gene in a human skin fibroblast cell line in which the paternal copy of the 15q11–q13 region was deleted. Moreover, intraperitoneal injection of UNC0642 significantly improved growth and survival in a mouse deletion model of PWS. Remarkably, the expression of *Snrpn* and *Snord116* was detectable in the brain and liver. To investigate the mechanisms that mediate the effects of UNC0642, a combination of bisulfite genomic sequencing and ChIP was carried out and a selective reduction of the demethylation of histone H3 lysine 9 (H3K9me2) at PWS-IC, without changing DNA methylation was demonstrated. This epigenetic modification is associated with a more open chromatin across the 15q11–q13 imprinted region [66]. This is an example of a principle for an epigenetics-based therapy.

## 3. Conclusions and Perspectives

The understanding of epigenetics is no longer limited to basic science. There is now a growing consciousness in the clinical field that having the correct pattern of epigenetic signatures is critical for the development of a normal phenotype. Recent research has focused on the role of epigenetic modifications at the GH-IGF-I axis. In this review, we discuss the progress in the understanding of the regulation of gene expression of the GH-IGF-I axis and the significant insights gained by recent research. If epigenetic mark patterns in the GH-IGF1 axis are not properly established or maintained during the fetal period, conditions as diverse as short stature, hypertension, T2DM, and cardiovascular disease may appear. Current understanding of DNA methylation and histone modifications that occur during fetal life is increasing, but much research is still required before translating these findings into clinical practice. Additionally, the uncovering of such epigenetic signatures would be crucially important in the exploration of therapeutic targets. In the following years research of the GH-IGF-I axis will allow for integration between the understanding of these epigenetic modifications and the development of forms of adjuvant therapies in the management of diverse growth-related conditions. The future paths for research in this area are, however, still not totally clear. It must also consider an emerging field that enhances the degree of complexity such as the contribution of noncoding RNA species, including microRNAs and long noncoding RNAs, in post-transcriptional regulation of gene expression in the GH-IGF-I axis and its deregulation in several growth-related epigenetic disorders. To this, we must add the epigenetic regulation of RNA (i.e., RNA methylation) and chromatin remodeling, which involves the repositioning or removal of nucleosomes on DNA, brought about by chromatin remodeling complexes (i.e., the SWI/SNF complex),

A promising field that can increase our knowledge of the regulation of gene expression of the GH-IGF-I can be found in emerging technologies of epigenetic editing. For example, the use of clustered regularly interspaced short palindromic repeats (CRISPR)/Cas9 technology and transcription activator–like effectors (TALEs) may demonstrate the causal role of epigenetic modifications in regulating chromatin conformation, gene transcription, and cellular phenotype by directing a particular epigenetic regulator to a specific genomic location [67,68]. Another promising field is in silico modeling, which can speed up research for innovative epigenetic therapeutic approaches to treat growth-related disorders, plot them to clinical estimates, and further our understanding of these conditions [3]. Finally, induced pluripotent stem cell (iPSC) disease models hold great promise for exploring pathophysiological mechanisms in clinically relevant human tissue and for drug screening/repurposing in a personalized manner [69].

## Abbreviations

ChIP	Chromatin ImmunoPrecipitation
*Cish*	Cytokine Inducible SH2 Containing Protein gene [not *Homo sapiens* (human)]
DNMT	DNA Methyl Transferase
ECD	Extracellular Domain
EHMT2	Euchromatic Histone Lysine *N*-Methyltransferase 2
FDA	US Food and Drug Administration
GH	Growth Hormone
GHR	Growth Hormone Receptor
GHRE	Growth Hormone Response Element
H4Kac	lysine acetylation at histone 4
H3K4	Mono-methylation of lysine 4 on histone H3
H3K9me3	trimethylation (me3) of lysine 9 on histone H3
H3K27me3	trimethylation (me3) of lysine 27 on histone H3
HDAC	Histone Deacetylase
ICD	Intracellular Domain
*IGF1*	Insulin-like Growth Factor I gene [Homo sapiens (human)]
*Igfals*	Insulin Like Growth Factor Binding Protein Acid Labile Subunit gene [not Homo sapiens (human)]
IGF-I	Insulin-like Growth Factor-I (protein)
IN2GHRE	Intron 2 Growth Hormone Response Element
IUGR	Intrauterine Growth Retardation
JAK2	Janus-Family Tyrosine Kinase-2 (JAK2)
miRNA	microRNA
PWS	Prader-Willi Syndrome
PWS-IC	Prader-Willi Syndrome-Imprinting Centre
rhGH	recombinant human Growth Hormone
SGA	Small for Gestational Age
*SNRPN*	Small Nuclear Ribonucleoprotein N Polypeptide gene (*Homo sapiens*)
*Socs2*	Suppressor of cytokine signaling 2 gene (not *Homo sapiens*)
*Spi2.1*	Transcription factor PU.1 gene (not *Homo sapiens*)
STAT	Signal Transducers and Activators of Transcription
T2DM	Type 2 Diabetes Mellitus
TCD	Transmembrane Domain
